# Risk Factors for Severe COVID-19 and Hospital Admission in Patients With Inborn Errors of Immunity - Results From a Multicenter Nationwide Study

**DOI:** 10.3389/fimmu.2022.835770

**Published:** 2022-02-28

**Authors:** Tomas Milota, Marta Sobotkova, Jitka Smetanova, Marketa Bloomfield, Jana Vydlakova, Zita Chovancova, Jiri Litzman, Roman Hakl, Jiri Novak, Ivana Malkusova, Jana Hanzlikova, Dalibor Jilek, Beata Hutyrova, Vitezslav Novak, Irena Krcmova, Anna Sediva, Pavlina Kralickova

**Affiliations:** ^1^Department of Immunology, Second Faculty of Medicine Charles University and Motol University Hospital, Prague, Czechia; ^2^Department of Paediatrics, First Faculty of Medicine, Charles University in Prague, Prague, Czechia; ^3^Department of Clinical and Transplant Immunology, Institute for Clinical and Experimental Medicine, Prague, Czechia; ^4^Department of Allergology and Clinical Immunology, Faculty of Medicine, Masaryk University and St Anne’s University Hospital in Brno, Brno, Czechia; ^5^Center for Clinical Immunology, Hospital Ceske Budejovice, Ceske Budejovice, Czechia; ^6^Department of Immunology and Allergology, Faculty of Medicine and Faculty Hospital in Pilsen, Charles University in Prague, Pilsen, Czechia; ^7^Department of Allergology and Clinical Immunology, Institute of Health in Usti nad Labem, Usti nad Labem, Czechia; ^8^Department of Allergology and Clinical Immunology, University Hospital in Olomouc, Olomouc, Czechia; ^9^Department of Immunology and Allergy, Institute of Health in Ostrava, Ostrava, Czechia; ^10^Institute of Clinical Immunology and Allergy, Faculty of Medicine in Hradec Kralove, University Hospital Hradec Kralove, Charles University, Hradec Kralove, Czechia

**Keywords:** inborn errors of immunity, COVID-19, SARS-CoV-2, risk factors, mortality, hospital admission

## Abstract

Despite the progress in the understanding how COVID-19 infection may impact immunocompromised patients, the data on inborn errors of immunity (IEI) remain limited and ambiguous. Therefore, we examined the risk of severe infection course and hospital admission in a large cohort of patients with IEI. In this multicenter nationwide retrospective survey-based trial, the demographic, clinical, and laboratory data were collected by investigating physicians from 8 national referral centers for the diagnosis and treatment of IEI using a COVID-19-IEI clinical questionnaire. In total, 81 patients with IEI (including 16 with hereditary angioedema, HAE) and confirmed SARS-CoV-2 infection were enrolled, and were found to have a 2.3-times increased (95%CI: 1.44–3.53) risk ratio for hospital admission and a higher mortality ratio (2.4% *vs.* 1.7% in the general population). COVID-19 severity was associated with the presence of clinically relevant comorbidities, lymphopenia, and hypogammaglobulinemia, but not with age or BMI. No individuals with HAE developed severe disease, despite a hypothesized increased risk due to perturbed bradykinin metabolism. We also demonstrated a high seroconversion rate in antibody-deficient patients and the safety of anti-spike SARS CoV-2 monoclonal antibodies and convalescent plasma. Thus, IEI except for HAE, represent significant risk factors for a severe COVID-19. Therefore, apart from general risk factors, immune system dysregulation may also be involved in the poor outcomes of COVID-19. Despite the study limitations, our results support the findings from previously published trials.

## Introduction

Inborn errors of immunity (IEI) are a heterogeneous group of rare disorders characterized by impaired immune system function, manifesting as increased susceptibility to infections and a broad spectrum of non-infectious complications including autoimmunity, autoinflammatory diseases, allergy, and/or malignancy (1, 2). In contrast to the general population, IEI predispose patients to severe, chronic, and recurrent infections, usually with complicated courses (3). Therefore, the novel coronavirus SARS-CoV-2 raised new concerns in patients with IEI patients. This single stranded mRNA virus was originally described in a patient with acute respiratory failure in the Chinese city of Wuhan; the disease was later named COVID-19 (4). To date, more than 250 million cases and 5 million deaths have been reported to be caused by COVID-19 (5). The main clinical features comprise fever, cough, malaise, fatigue, sore throat, and dyspnea that may rapidly progress to acute lung injury with acute respiratory failure (6, 7). Higher age (> 75 years), severe obesity, male sex, arterial hypertension, and cardiovascular or respiratory tract disease were determined as the main risk factors for a severe disease course and poor outcome (8, 9). Higher age (> 65 years) was also associated with an increased risk of reinfection (10), which is further emphasized by the emergence of novel virus variants (11). Complicated COVID-19 infection with increased hospital admission and mortality rates compared to the general population was also described in immunocompromised patients such as those with cancer or solid organ transplants. However, many of these patients had other risk factors (higher age, significant comorbidities) associated with worse outcomes. Interestingly, children who underwent hematopoietic stem cell transplantation showed a similar risk ratio for severe COVID-19 compared to the general population (12). Despite the progress in understanding how COVID-19 may impact patients with IEI, studies focused on the clinical presentation and outcome of COVID-19 in these patients remain limited, and present ambiguous results (13–17) that may reflect inter-population differences. Therefore, we initiated a retrospective multicenter study focused on the clinical presentation and outcome of COVID-19 in patients with IEI. The study included patients with hereditary angioedema (HAE) due to C1 inhibitor deficiency, as the bradykinin overproduction in the kallikrein-kinin cascade was proposed as a possible mediator involved in the respiratory complications of COVID-19 infection and, as such, a risk factor for severe COVID-19.

## Methods

### Study Design

**Figure 1:**
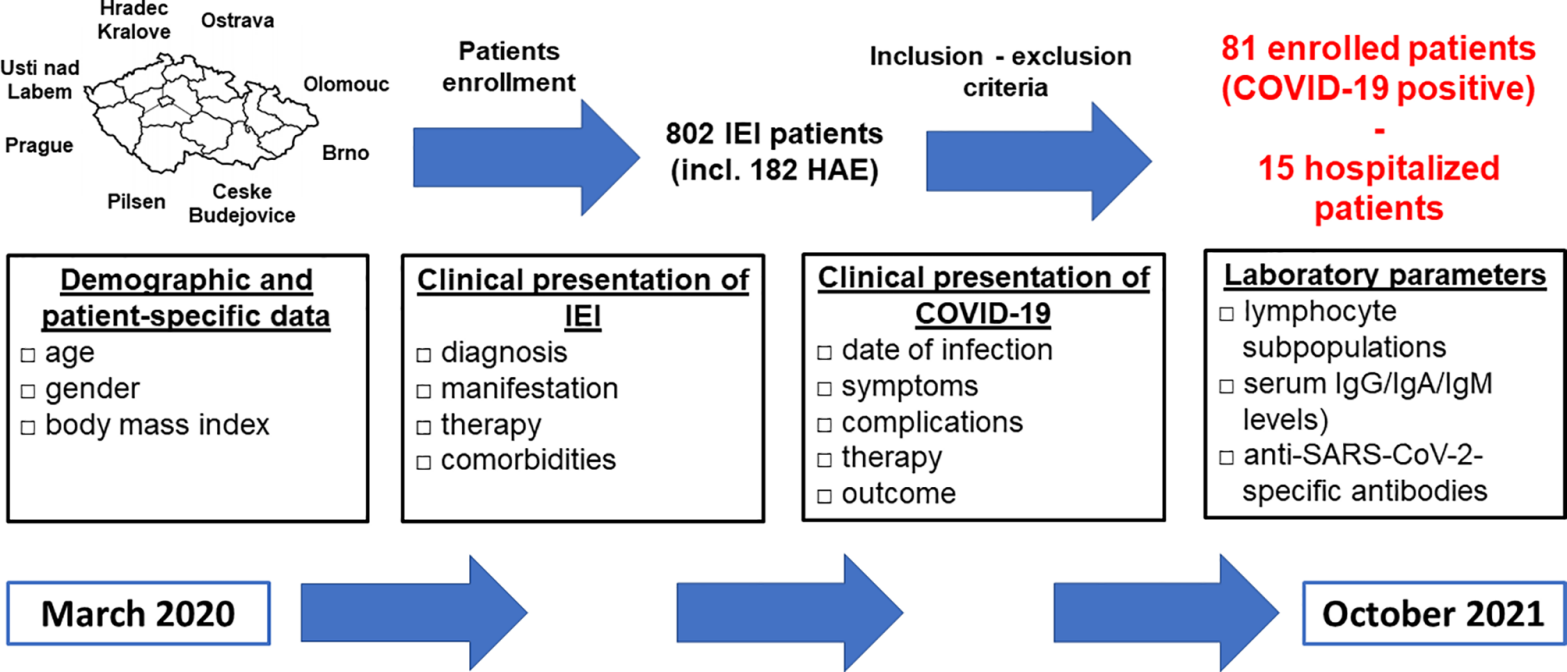
Study design - participating centers, enrollment process, study (data collection) period and extent (characteristics) of collected data are shown (IEI, inborn errors of immunity; HAE, hereditary angioedema; Ig, immunoglobulin).

The study was designed as a multicenter retrospective survey-based study and was conducted for the period from March 2020 to October 2021. Data were obtained from 8 national referral centers for the diagnosis and treatment of IEI including patients with hereditary angioedema (HAE), who were evaluated separately. The study only included patients fulfilling the following inclusion criteria: 1) fulfilled diagnostic criteria for IEI, 2) PCR SARS-CoV2 positivity, the testing was indicated according to the recommendations of the healthcare authorities for the general population, 3) patient´s consent provided before inclusion. The study design is summarized in **Figure 1**. This study was approved by Ethics Committee of the University Hospital in Hradec Kralove, the Czech Republic.

### Data Collection

Demographic and patient-specific data (gender, age, body mass index - BMI), the clinical presentation of IEI (diagnosis, manifestation, and therapy), comorbidities, the clinical presentation of COVID-19 (date of infection, symptoms, disease-related complications, therapy, and outcome), and laboratory parameters (lymphocyte subpopulations, serum IgG/IgA/IgM levels) as close as possible to the time of COVID-19 diagnosis were collected. Patients were classified based on the European Society for Immunodeficiency diagnostic criteria (18) and/or genetically confirmed diagnosis. Patients with humoral immunodeficiency and excluded secondary causes of hypogammaglobulinemia who did not meet diagnostic criteria were classified as unclassified primary antibody deficiency (unPAD). Subsequently, seroconversion was assessed as the presence of anti-SARS-CoV-2-specific IgG/IgA/IgM antibodies after recovery, the patients who received anti-spike SARS-CoV-2 monoclonal antibodies were excluded. The specific antibodies were detected using chemiluminescent immunoassays (LIAISON SARS-CoV-2 S1/S2 IgG, DiaSorin, Saluggia, Italy) or enzyme-linked immunosorbent assays (Elisa SARS-CoV-2 IgG, EUROIMMUN, Lübeck, Germany; COVID-19 RBD IgG, TestLine Clinical Diagnostics Ltd., Brno, Czech Republic). All data were collected by investigating physicians using a uniform COVID-19-IEI clinical questionnaire. The patients were divided into 3 groups - 1) asymptomatic, 2) symptomatic with mild/moderate disease, 3) symptomatic with a severe disease course (requiring hospital admission). The results were compared with data from the general population, obtained from the Ministry of Health, the Czech Republic (19) and the Czech Statistical Office (20).

### Statistical Analysis

Mean and standard deviation (SD) were calculated for the continuous data (age, BMI, laboratory parameters). The statistically significant differences of the means were assessed by Mann-Whitney test for unpaired data with non-normal distribution. The normality was tested using Shapiro-Wilk normality test. Proportion, risk ratio (relative risk, RR) and 95% confidence interval were calculated for attributive data (gender, prevalence, hospital admission, risk factors). The statistically significant differences of the proportions were evaluated by Chi-squared test. Statistical significance was reached when p value < 0.05. Statistical analysis was performed using GraphPad Prism 8 (GraphPad Software, San Diego, CA, USA)

## Results

### Cohort Characteristics

**Figure 2:**
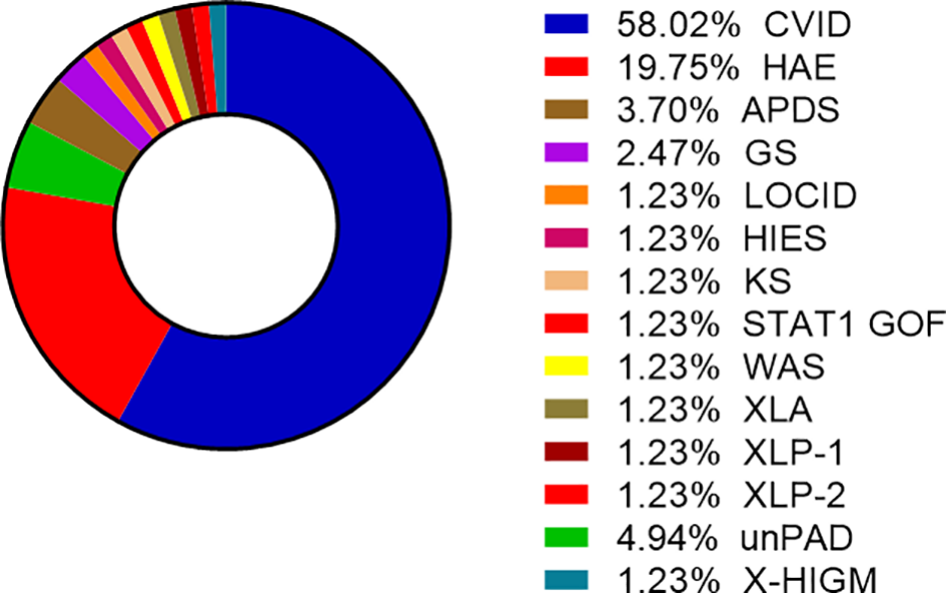
Proportion of enrolled patients (n= 81) according to the type of Inborn errors of immunity (IEI), other IEI include GS, LOCID, HIES, KS, STAT-1 GOF, WAS, XLA, XLP1/2 (CVID, common variable immunodeficiency; HAE, hereditary angioedema; APDS, activated phosphoinositide 3-kinase δ syndrome; GS, Good´s syndrome; LOCID, late-onset combined immunodeficiency; HIES, hyper IgE syndrome; KS, Kabuki syndrome; CMC, STAT-1 gain-of-function chronic mucocutaneous candidiasis; WAS, Wiskott-Aldrich syndrome; XLA, X-linked agammaglobulinemia; XLP-1/2, X-linked lymphoproliferative syndrome type 1/2; unPAD, unclassified hypogammaglobulinemia; X-linked hyper IgM syndrome).

**Figure 3:**
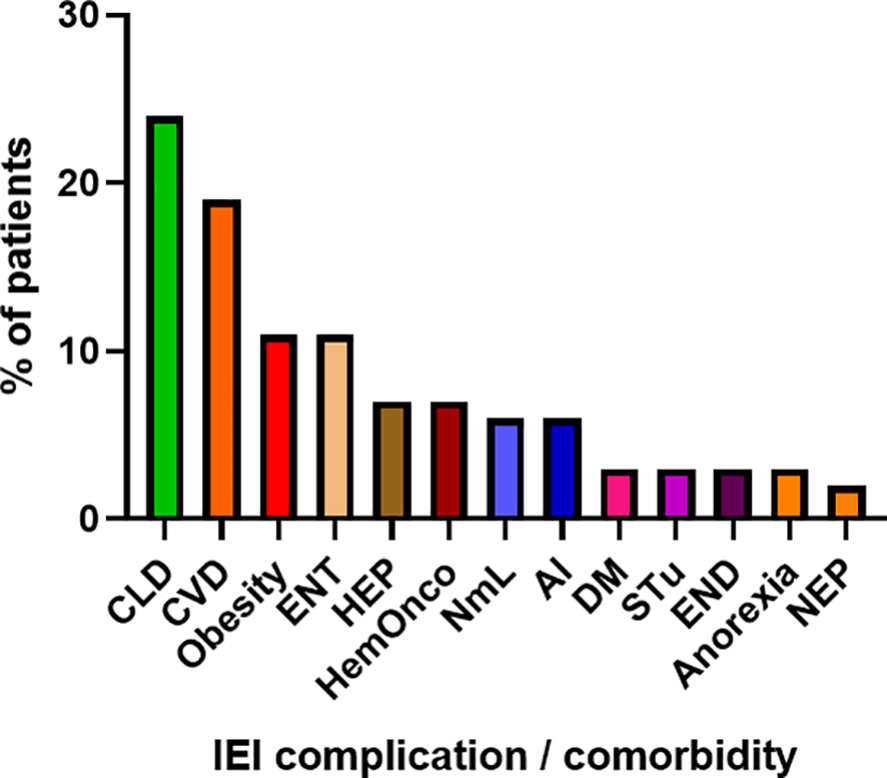
Proportion of IEI (Inborn erorrs of immunity)-related complications and comorbidities in enrolled patients (n = 81). (CLD, chronic lung disease; CVD, cardiovascular disease; ENT, enteropathy; HEP, hepatopathy; HemOnco, hematologic malignancy; NmL, non-malignant lymphoproliferation; AI, autoimmunity; DM, diabetes mellitus; STu, solid tumor; END, endocrinopathy; NEP, nephropathy).

**Figure 4:**
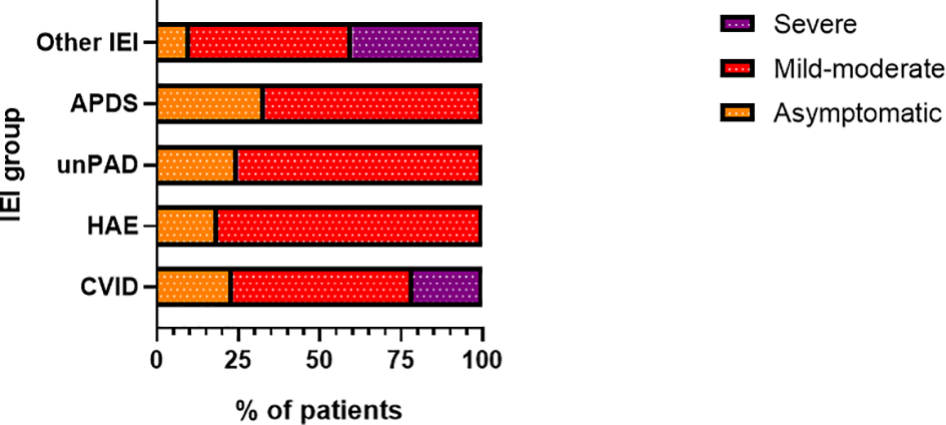
Proportion of patients (n= 81) according to disease severity in particular inborn erorrs of immunity (IEI) categories (CVID, common variable immunodeficiency, n = 47/81; HAE, hereditary angioedema, n = 16/81; unPAD, unclassified primary antibody deficiency; n = 4/81; APDS, Activated phosphoinositide 3-kinase δ syndrome, n = 3/81; IEI, n = 11/81).

COVID-19 infection was diagnosed in 81 patients with IEI (47 females, 34 males) including 16 cases of HAE in a multicenter cohort of 805 patients with IEI (including 182 HAE patients) followed up at 8 national referral centers in the Czech Republic, indicating an incidence of 10.1%, compared to that of 16.6% in the general population (1.77 million cases of COVID-19 infection in the 10.69 million population). Interestingly, these data suggest a significantly (p ˙ 0.0001) higher risk (RR= 1.66, 95%CI: 1.33–1.99) of COVID-19 infection in the general population. The risk of infection remained almost unchanged when HAE was excluded from the analysis (RR= 1.59, 95%CI: 1.27 – 2.00). The mean patient age was 42.41 years (± 16.07 SD) and mean BMI was 25.88 kg/m^2^ (± 6.43 SD). The largest proportion of infection among patients with IEI (COVID-IEI) was observed in common variable immunodeficiency (CVID: 58%, n = 47/81), followed by that in HAE (19.8%, n= 16/81), unPAD (5%, n = 4/81), activated PI3K delta syndrome (APDS: 3.7%, n = 3/81), and Good´s syndrome (GS: 2.5%, n = 2/81). Other IEI were represented by only one patient, including late-onset combined immunodeficiency (LOCID), hyper IgE syndrome (HIES), Kabuki syndrome (KS), STAT-1 gain-of function chronic mucocutaneous candidosis (CMC), Wiskott-Aldrich syndrome (WAS), X-linked agammaglobulinemia (XLA), X-linked lymphoproliferative syndrome type 1 and type 2 (XLP-1, 2), and X-linked hyper IgM syndrome (**Figure 2**). The COVID-IEI cohort included 47 (58.5%) females and 34 males (41.5%). The most common comorbidities and IEI-related complications reported in COVID-IEI patients were chronic lung (29.6%, n = 24/81) and cardiovascular diseases (23.5%, n = 19/81), followed by enteropathy (13.6%, n =11/81), obesity (13.6%, n = 11/81), hepatopathy (8.6%, n = 7/81), hematologic malignancy (8.6%, n = 7/81), non-malignant lymphoproliferation (7.4%, n = 6/81), and serious autoimmunity (7.4%, n = 6/81) (complete list in **Figure 3**). IRT was indicated in 75.3% of patients (n = 61/81) including all CVID and APDS patients among others. Intravenous (10% IgG solutions), subcutaneous (10%, 16.5%, 20% resp. IgG solutions) and facilitated subcutaneous remedies (10% IgG solutions) were used. Nineteen patients (23.4%) were using immunosuppressive therapy prior to COVID-19 infection. Glucocorticosteroids (GC: 73.7%, n = 14/19), direct PI3K inhibitors (15.8%, n = 3/19), and rituximab (10.5%, n = 2/19, in one case as a component of combined chemotherapy along with cyclophosphamide, doxorubicin, and prednisone) were the most frequent immunomodulatory drugs. Asymptomatic infection was observed in 21% (n = 17/81) of the patients. The main manifestations in symptomatic patients included fever (45.3%, n = 29/64), dyspnea (31.3%, n = 20/64), cough (26.6%, n = 17/64), flu-like symptoms (26.6%, n = 17/64), loss of smell/taste (26.6%, n = 17/64), upper respiratory tract symptoms (25%, n = 16/64), fatigue (23.4%, n = 15/64), and headache (9.4%, n = 6/64). All the reported symptoms are summarized in **Figure 4**. Pneumonia developed in 19 patients, in whom diagnosis was confirmed by imaging methods. Ischemic (heart attack) or thromboembolic (pulmonary vein embolism) events occurred in two patients. Two patients (APDS and XLP-2) were infected by SARS-CoV-2 despite appropriate vaccination. Both of them manifested with the flu-like and upper respiratory tract infection symptoms. None of them required hospital admission. The anti-spike SARS-CoV-2-specific monoclonal antibody, bamlanivimab, was used in 1 hospitalized patient whereas convalescent plasma was indicated in 4 patients. Two other applications of bamlanivimab and casirivimab/imdevimab were administered in an outpatient regimen. None of these patients required hospital admission. Generally, administration was not associated with any adverse event. None of the IEI patients was fully vaccinated (completed vaccination schedule) prior to infection due to limited availability of the vaccines at the beginning of the COVID-19 pandemic. Only a single patients received 1 dose from 2-dose vaccine scheme. The remaining patients were vaccinated after infection.

### Risk of Severe Course for COVID-19 and Hospital Admission

**Figure 5:**
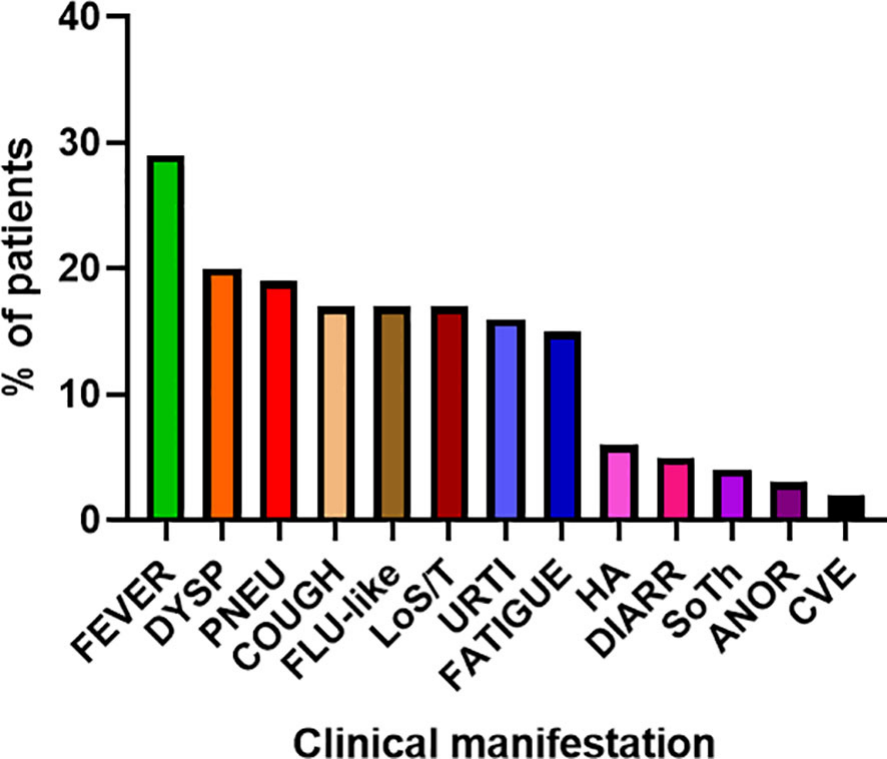
Main clinical manifestations of COVID-19 in enrolled patients (n= 81) (DYSP, dyspnea; PNEU, pneumonia; LoS/T, loss of smell/taste; URTI, upper respiratory tract infections; HA, headache; DIARR, diarrhea; SoTh, sore throat; ANOR, Anorexia; CVE, cardiovascular event).

Overall, 15 patients with IEI required hospital admission, corresponding to 18.5%. In contrast to the risk for infection, patients with IEI showed a significantly (p = 0.0011) increased risk (RR) for hospital admission, which was 2.3-times higher (95%CI: 1.44 - 3.53). The risk for hospital admission further increased when HAE patients were excluded (RR= 2.91, 95%CI: 1.83 – 4.37, p < 0.0001). In the general Czech population, the total of 139 600 COVID-19 positive individuals needed hospital admission by the time of the study period, equaling 7.9%. In our study, the highest number of hospital admissions was reported in amongst the CVID patients. In total, 10 out of 47 patients with CVID (21.3%) were hospitalized. This corresponds to 2.68 RR (95%CI: 1.51 – 4.4). Other patients requiring hospital admission were diagnosed with LOCID, GS, KS, HIES, and WAS (one patient in each group). No hospitalization was documented in the remaining groups (**Figure 5**). The major symptom leading to hospital admission was dyspnea (66.7%, n = 10/15). Two patients experienced newly diagnosed atrial fibrillation and heart attack. Eight patients (53.3%) required intensive care, the remaining patients were hospitalized at standard care departments. High-flow nasal oxygen was needed in 5 patients (33.3%) and artificial ventilation was required in 2 patients (13.3%). Other medications (mono- or combined therapy) included the use of antibiotics (46.7%, n = 7/15), GC (40%, n = 6/15), and antivirotics such as remdesivir and/or favipiravir (33.3%, n = 5/15). Two patients with other significant comorbidities died due to severe complications. This corresponds to a mortality rate of 2.4% in the COVID-IEI cohort and 13.3% among hospitalized patients. In contrast, 1.7% mortality was reported in the general population (30.90 thousand COVID-19 associated deaths out of 1.766 million infected individuals).

### Risk Factors Associated With Hospital Admission

We did not find statistically significant differences in age (45.33 ± 15.32 SD *vs.* 41.36 years ± 16.57 SD), gender ratio, or BMI (body mass Index: 27.17 ± 8.52 *vs.* 25.59 kg/m^2^ ± 5.90 SD) value between hospitalized and non-hospitalized patients. However, we found significant differences in the absolute number of total lymphocyte counts (1.18 E9/L ± 0.84 SD *vs.* 1.75 E9/L ± 0.84 SD, p = 0.016), T cells (CD3+) specifically (0.92 E9/L ± 0.58 SD *vs.* 1.40 E9/L ± 1.0 SD, p = 0.03). The differences in the CD4+ and CD8+ T cells were not statistically significant. Hospitalized individuals also had a significantly lower number of B cells (CD19+: 0.068 E9/L ± 0.06 SD *vs.* 0.17 E9/L ± 0.15 SD, p = 0.004), NK cells (CD3- CD56+: 0.12 E9/L ± 0.16 SD *vs.* 0.2 E9/L ± 0.17 SD, p = 0.01) along with serum levels of IgA (0.3 g/L ± 0.64 SD *vs.* 0.76 g/L ± 1.14 SD, p = 0.04) and IgM (0.15 g/L ± 0.19 SD *vs.* 0.50 g/L ± 0.66 SD, p = 0.0095) in contrast to IgG values, which were comparable between patient groups (**Table 1**). When looking separetely at CVID sub-cohort, representing the largest IEI group, we found significant differences in B cells counts only (0.05 E9/L ± 0.06 SD vs. 0.17 E9/L ± 0.13 SD). Analyzing the CVID patients receiving IRT, we did not revealed significant differences between serum IgG trough levels of the hospitalized (6.65 IgG ± 2.1 SD) and non-hospitalized CVID individuals (6.87 IgG ± 2.5 SD). Regarding comorbidities and IEI-related complications, chronic lung disease (53.3%, n = 8/15) and cardiovascular diseases (33.3%, n = 5/15) were the most prevalent. Further, more than half of the patients (60%, n = 9/15) had at least two comorbidities/complications (**Table 2**).

**Table 1:** Laboratory parameters associated with risk of hospital admission.

Parameter	Hosp+	Hosp-	p-value
Lymphocyte count (10E9/L, ± SD)	1.18 ± 0.84	1.75 ± 0.84	0.016
T cell count (10E9/L, ± SD)	0.92 ± 0.58	1.40 ± 1.0	0.03
B cell count (10E9/L, ± SD)	0.06 ± 0.06	0.17 ± 0.15	0.004
NK cell count (10E9/L, ± SD)	0.12 ± 0.16	0.2 ± 0.17	0.01
Serum IgA (g/L, ± SD)	0.3 ± 0.64	0.76 ± 1.1	0.04
Serum IgM level (g/L, ± SD)	0.15 ± 0.19	0.51 ± 0.66	0.01

(SD, standard deviation; Hosp+, hospitalized patients; Hosp-, non-hospitalized patients).

**Table 2:** Characteristics of hospitalized patients.

Diagnosis	Gender	Age (yrs.)	BMI (kg/m^2^)	Comorbidity	Immuno-suppressants	IRT	Treatment level	Oxygen therapy	Other treatment	Outcome
CVID	M	51	35.1	AH, DM, obesity	0	Yes	ICU	HFNO	REM	resolved
CVID	F	25	22.9	CLD	GC, RTX	Yes	SC	N/R	GC, CP, SMA	resolved
CVID	F	59	19.0	Absent	0	Yes	SC	N/R	Symptomatic	resolved
CVID	F	32	49	Obesity	0	Yes	SC	NC	GC, ATB, REM, CP	resolved
CVID	F	50	34.2	AH, CLD, obesity	GC	Yes	ICU	AV	ATB	died
CVID	M	27	12.9	AH, CLD, anorexia	0	Yes	SC	NC	ATB	died
CVID	F	65	22.6	CLD, HEP, THRO	0	Yes	SC	N/R	GC	resolved
CVID	F	40	24.4	HEP, CED, LYMPH	GC	Yes	ICU	HFNO	GC, ATB	resolved
CVID	F	46	30.1	CLD, LYMPH, obesity	GC	Yes	SC	NC	GC, ATB, REM	resolved
CVID	F	69	26	CLD	0	Yes	ICU	HFNO	GC, ATB	resolved
LOCID	F	34	24.3	CLD, SPLE	0	Yes	ICU	HFNO	ATB, REM, FAV, CP	resolved
GS	M	68	25.8	AH, DM, PCa	0	Yes	ICU	HFNO	CP	resolved
KS	M	23	32.9	NEPH, HPIT, CHD, AIHA	0	Yes	ICU	AV	Symptomatic	resolved
HIES	F	46	28.7	CLD	0	Yes	ICU	NC	REM	resolved
WAS	M	45	19.8	NEPH	GC	Yes	SC	N/R	Symptomatic	resolved

(IRT, immunoglobulin replacement therapy; yrs, years; BMI, body mass index; m, male; f, female; CVID, common variable immunodeficiency; LOCID, late-onset combined immunodeficiency; GS, Good´s syndrome; KS, Kabuki syndrome; HIES, hyper IgE syndrome; WAS, Wiskott-Aldrich syndrome; AH, arterial hypertension; DM, diabetes mellitus; CLD, chronic lung disease; HEP, hepatopathy; THRO, thrombocytopenia; CED, celiac disease; LYMPH, lymphadenopathy; SPLE, splenomegaly; PCa, prostate cancer; NEPH, nephropathy; HPIT, hypopituitarism; CHD, congenital heart defect; AIHA, autoimmune hemolytic anemia; SC, standard care; ICU, intensive care unit; N/R, not required; NC, nasal cannula; HFNO, high-flow nasal oxygen; AV, artificial ventilation; ATB, antibiotics; REM, remdesivir; FAV, favipiravir; GC, glucocorticoids; CP, convalescent plasma; SMA, anti-spike SARS-CoV-2 specific monoclonal antibodies; RTX, rituximab).

### The Seroconversion Rate Upon Recovery

Seroconversion was assessed in 59.3% (n = 48/81) of the enrolled patients after 2.67 months (mean time, range: 1 – 7 months) from recovery. The patients who were exposed to anti-spike SARS-CoV-2 monoclonal antibodies and/or convalescent plasma were excluded from the seroconversion analysis. Anti-SARS-CoV-2 specific antibodies were detected in 68.8% (n= 33/48) of the recovered patients, including 7/9 (77.8%) asymptomatic patients, 21/31 (67.7%) outpatients, and 5/8 (62.5%) hospitalized patients. Surprisingly, a high seroconversion rate of 76% (n = 19/25) was also observed in patients with CVID patients who were expected to show heavily impaired production of antigen-specific antibodies.

## Discussion

IEI are rare diseases that predispose patients to increased risk of infections and their severe course. Therefore, patients with IEI were also regarded as a risk population for severe COVID-19. However, data from different studies indicated ambiguous conclusions. A multicenter study from Israel showed a lower incidence of COVID-19 infection among patients with IEI (1.2%) compared with that in the general population (2.5%). These data are consistent with our results, showing a 1.66-fold higher risk of COVID-19 infection in the general population of the Czech Republic. Here, the reported incidence was 10.1% and 16.6% in the IEI cohort and the general population, respectively. The risk of infection remained almost unchanged when HAE was excluded from the analysis. Nevertheless, these results may be biased due to the cohort size, thus they should be interpreted carefully. Several factors may affect the prevalence, such as tighter adherence of IEI patients to preventive measures, including stricter infection-avoidance behavior or early vaccination, compared to the general population. However, the authors reported a high number of asymptomatic patients (n = 6/20) and a only single case of pneumonia (13), which was in contrast to the United Kingdom Primary Immunodeficiency Network registry-based study. In this study, we observed 20% (n = 12/60) infection-fatality ratio and a 37.5% inpatient mortality rate. A higher age and comorbidities such as chronic lung disease, cardiovascular disease, and diabetes were clinically important risk factors. Poor outcome was also associated with low lymphocyte count (14). Similarly, we observed a 2.3-fold increased risk ratio for hospital admission, which was the highest rate for patients with CVID patients (21.3%). Hospital admission was also associated with higher mortality (13.3% *vs.* 2.4% in the entire IEI cohort). However, we could not prove the impact of either age or BMI. Apart from low lymphocyte counts, we observed significant differences between hospitalized and non-hospitalized patients with IEI in the number of T, B, and NK cells along with reduced serum levels of IgA and IgM. Nevertheless, we found significant differences in B cells counts only when looking at CVID sub-cohort separately. Surprisingly, we did not observe any differences in serum IgG levels (measured as trough levels in patients on regular immunoglobulin substitution). A high mortality rate was also observed in a robust international multicenter retrospective web-based survey, reaching 10% (n = 9/94) but all adult patients had other pre-existing comorbidities such as a higher age, chronic lung, cardiovascular, or chronic renal disease and others. Hospital admission was required in 63% of the patients (n = 59–94). An asymptomatic course of SARS-CoV-2 infection was reported in only 11% of the patients with IEI (16). Based on these results, we assumed an important role of IEI itself, which is also supported by other studies (15, 17, 21) and meta-analyses (22). Additionally, anti-spike SARS-Cov2 monoclonal antibodies and convalescent plasma seem to be safe and possibly effective treatment options for patients with IEI. However, these observations need to be verified in clinical trials. More than half of the patients in each patient group (asymptomatic, outpatient, hospitalized) also exhibited humoral seroconversion after recovery. A high seroconversion rate was also observed in patients with CVID who show impaired production of antigen-specific antibodies. High seroconversion rate was later reported in IEI patients, including severe antibody deficiencies, following SARS-CoV-2 vaccination and may may provide safe and effective solution for IEI patients. However, the immunogenicity varies between the specific type of IEI (23).

With regard to patients with HAE, they showed neither SARS-CoV-2-associated hospital admission nor death despite previously proposed role of the bradykinin overproduction in the kallikrein-kinin cascade as a possible co-mediator of COVID-19-related pulmonary complications (24–26). However, three cohorts of patients with HAE who contracted COVID-19 did not confirm this hypothesis (17, 27). Thus, our observations support these findings. This could be also explained by a small number of risk factors for severe COVID-19 disease.

There are several limitations of this study. These primarily include the retrospective study design, survey-based data collection, lower number of enrolled patients, and the heterogeneity of the cohort. Therefore, the results should be interpreted carefully in the context of other studies. However, the strengths of this study include its nation-wide multicenter design including national referral centers for the management of patients with IEI. Our data also represent a substantial sample of Central European population with IEI.

## Conclusion

Our study presents the results from a multicenter nationwide retrospective survey-based study including 81 patients with IEI (including 16 with HAE) in whom SARS-CoV-2 infection was confirmed. We revealed a lower risk of COVID-19 infection but a higher risk ratio for hospital admission and mortality compared to that in the general population. A severe course of COVID-19 was reported mainly in patients with significant comorbidities. However, we also assume that immune system dysregulation contributed to these outcomes based on the findings of lymphopenia and hypogammaglobulinemia as the risk factors for severe course of the disease. The use of anti- spike SARS-Cov2 monoclonal antibodies and convalescent plasma provide safe and effective therapeutic options for patients with IEI. In line with the previous studies, we did not observe an increased risk of COVID-19 in patients with HAE despite the expected role of bradykinin overproduction in COVID-19 associated respiratory complications. COVID-19 infection also resulted in a high seroconversion rate after recovery even in antibody-deficient patients.

## Data Availability Statement

The raw data supporting the conclusions of this article will be made available by the authors, without undue reservation.

## Ethics Statement

The studies involving human participants were reviewed and approved by Ethics Committee of the University Hospital in Hradec Kralove, the Czech Republic. Written informed consent for participation was not required for this study in accordance with the national legislation and the institutional requirements.

## Author Contributions

TM and MS (main authors) made substantial contributions to the conception and design of the study, analysis and interpretation of data, drafted the manuscript. JS, MB, JV, ZC, JL, RH, JN, IM, JH, DJ, BH, VN, and IK (co-authors) made substantial contributions to the acquisition of data, analysis and interpretation. AS and PK (senior authors) made substantial contributions to the conception and design of the study, reviewed the manuscript critically for important intellectual content, gave final approval of the version to be published.

## Funding

The study was supported by the grant of the Czech Health Research Council nr. NV18-05-00162.

## Conflict of Interest

The authors declare that the research was conducted in the absence of any commercial or financial relationships that could be construed as a potential conflict of interest.

## Publisher’s Note

All claims expressed in this article are solely those of the authors and do not necessarily represent those of their affiliated organizations, or those of the publisher, the editors and the reviewers. Any product that may be evaluated in this article, or claim that may be made by its manufacturer, is not guaranteed or endorsed by the publisher.
